# Generation of a Functionally Distinct *Rhizopus oryzae* Lipase through Protein Folding Memory

**DOI:** 10.1371/journal.pone.0124545

**Published:** 2015-05-13

**Authors:** Atsushi Satomura, Kouichi Kuroda, Mitsuyoshi Ueda

**Affiliations:** 1 Division of Applied Life Sciences, Graduate School of Agriculture, Kyoto University, Sakyo-ku, Kyoto, Japan; 2 Japan Society for the Promotion of Science, Sakyo-ku, Kyoto, Japan; Louisiana State University Health Sciences Center, UNITED STATES

## Abstract

*Rhizopus oryzae* lipase (ROL) has a propeptide at its N-terminus that functions as an intramolecular chaperone and facilitates the folding of mature ROL (mROL). In this study, we successfully generated a functionally distinct imprinted mROL (mROL^imp^) through protein folding memory using a mutated propeptide. The mutated propeptide left its structural memory on mROL and produced mROL^imp^ that exhibited different substrate specificities compared with mROL^WT^ (prepared from the wild type propeptide), although the amino acid sequences of both mROLs were the same. mROL^imp^ showed a preference for substrates with medium chain-length acyl groups and, noticeably, recognized a peptidase-specific substrate. In addition, ROL^imp^ was more stable than mROL^WT^. These results strongly suggest that proteins with identical amino acid sequences can fold into different conformations and that mutations in intramolecular chaperones can dynamically induce changes in enzymatic activity.

## Introduction

Most lipases and proteases are produced as precursor forms with propeptides and are subsequently processed into mature enzymes [1–4]. Some propeptides function as inhibitors that temporarily inhibit the enzymatic activities of their mature enzymes during cellular transport [5]. In addition, some propeptides play an essential role in protein maturation. These propeptides function as chaperones and facilitate the correct folding of their mature enzymes [6, 7]. Because propeptides are covalently bound to the mature enzyme, they are called “intramolecular chaperones,” distinct from “intermolecular chaperones.” Once the protein has been folded into its mature form, propeptides are removed by autolysis or exogenous proteases [5, 8].

In our previous study, we demonstrated that mutations in the propeptide of carboxypeptidase Y (CPY) generated a functionally distinct, mature CPY [9]. The mature form of CPY generated with the mutated propeptide had the same amino acid sequence as mature CPY prepared from the wild type propeptide, as both the wild type and mutated propeptides were completely cleaved off. This phenomenon, where the modified enzyme retains the “folding memory” of the mutated propeptide even after the propeptide digestion, has been called “protein folding memory [9]”. “Protein folding memory” contradicts Anfinsen’s dogma (Nobel Prize in 1972), which postulates that the structure of a protein is determined only by its amino acid sequence and that the native structure is a unique and most stable state. [10]. On the other hand, according to the “protein folding memory” theory, the structure of the mature enzyme is not only determined by the amino acid sequence but rather by the chaperoning function of the propeptide. Although “protein folding memory” is an attractive phenomenon, little is known about the underlying mechanism. “Protein folding memory” would have applications in the field of protein engineering and may be adopted to dynamically improve enzymatic functions through structural imprinting by propeptides.

*Rhizopus oryzae* lipase (ROL) is widely used in industrial applications [11]. ROL is initially produced as a precursor form, which comprises an N-terminal 69-amino acid propeptide and a 297-amino acid mature enzyme [12]. The propeptide of ROL (proROL) functions as an inhibitor and intramolecular chaperone for mature ROL (mROL) [13]. When ROL is produced in *Saccharomyces cerevisiae* and *Pichia pastoris*, proROL is cleaved by Kex2-like proteases, resulting in mROL [5, 14]. A previous study revealed that residues Ser20–Gly37 and Ser38–Glu57 in proROL were essential for the secretion and folding of mROL, respectively [6]. The active site of ROL is composed of the Ser242, Asp301, and His354 residues; the same three residues are also observed in the catalytic triad of serine proteases and peptidases [15]. Although there are only subtle differences between the active sites of fungal lipases and serine proteases, their substrate recognition mechanisms are different. The active centers of proteases and peptidases are exposed to the solvent, while those of lipases are not [16, 17]. The catalytic triads of lipases are buried under a short α-helix, called lid domain. Lid opening is accompanied by a concomitant structural change in the substrate-binding site that enables the binding of the substrate to the active site [18]. The differences in the activities and substrate recognition mechanisms between lipases and proteases may be partially attributed to the conformational characteristics of lipases. The conformation and activity of mROL would be altered by introducing mutations in an essential part of proROL for chaperoning function. This would allow conformational changes in the substrate-recognition domain of the mature protein and lead to the altered activity.

In this study, we demonstrated that mutations in proROL led to “protein folding memory” and generated a functionally distinct imprinted mROL (mROL^imp^) that had the same amino acid sequence as mROL prepared from wild-type proROL (mROL^WT^). Although mROL^imp^ and mROL^WT^ had the same amino acid sequences, mROL^imp^ showed higher lipase activity and stability compared with mROL^WT^, and recognized a peptidase-specific substrate.

## Materials and Methods

### Strain and materials

*Escherichia coli* strain DH5α [F^-^, *end*A1, *hsd*R17(r_k_^-^/m_K_^+^), *sup*E44, *thi-*1, λ^-^, *deo*R, *rec*A1, *gyr*A96, *pho*A, φ80d*lac*ZΔM15, Δ(*lac*ZYA-*arg*F)U169] (Toyobo, Osaka, Japan) was used as the host for recombinant DNA manipulation. *P*. *pastoris* strain GS115 [*his4*] (Life Technologies, CA, USA) was used as the host for protein production. *S*. *cerevisiae* strain BY4741/*sed1*Δ [*MAT* **a**, *his3*Δ1, *leu2*Δ0, *met15*Δ0, *ura3*Δ0] (EUROSCARF, Frankfurt, Germany) was used for the cell surface display of mROL^WT^ and mROL^imp^. *E*. *coli* transformants were grown in Luria-Bertani media (1% [w/v] tryptone, 0.5% [w/v] yeast extract, and 1% [w/v] sodium chloride) containing 50 μg/mL ampicillin. For protein production, *P*. *pastoris* transformants were pre-cultivated in buffered complex glycerol media (BMGY; 1% [w/v] yeast extract, 2% [w/v] peptone, 1.34% [w/v] yeast nitrogen base without amino acids, 4 × 10^−5^% [w/v] biotin, 1% [v/v] glycerol, and 100 mM potassium phosphate [pH 6.0]). To induce transcription, pre-cultivated transformants were grown in buffered complex methanol media (BMMY) (1% [w/v] yeast extract, 2% [w/v] peptone, 1.34% [w/v] yeast nitrogen base w/o amino acids, 4 × 10^−5^% [w/v] biotin, 0.5% [v/v] methanol, and 100 mM potassium phosphate [pH 6.0]). Yeast transformants were cultured in synthetic dextrose (SD) medium (0.67% [w/v] yeast nitrogen base w/o amino acids and 2% [w/v] glucose) supplemented with the appropriate amino acids.

### Construction of plasmids

The *ROL* gene was amplified from the pWRL2 [3] plasmid using F proROL c-Myc primer (5'- TCTGTCTTCGCTCGAGAACAAAAGTTGATTTCTGAAGAAGATTTGGTTCCTGTTTCTGGTAAATCTGG -3') and R mROL His-tag primer (5'- AAGGATCCCGGGGAATTAATGATGATGATGATGATGCAAACAGCTTCCTTCGTTGATATC -3'), and inserted into the pHIL-S1 plasmid (Life Technologies) for protein production by *P*. *pastoris*. The resulting plasmid, labeled pHIL-proROL-mROL, was composed of the *PHO1* secretion signal sequence, c-Myc-tag-encoding sequence, *ROL* gene, and His tag-encoding sequence. To construct the gene encoding the mutated propeptide, DNA fragments encoding the Val1–Met49 and Tyr56–Leu366 amino acid residues of ROL were amplified using the F proROL c-Myc primer and 3’ primer (5'- CATGTAGTAAGGTTCAGCTTGAAG -3'), and 5’ primer (5'- TCCCATGGTGGCAACCTGAC -3') and R mROL His-tag primer, respectively. These DNA fragments were fused with a DNA fragment (5'- TTCAAGCTGAACCTTACTACATGGTTGATGATGATGATAAATATGAGTCCCATGGTGGCAACCTGACATC -3') encoding the mutated sequence (VDDDDK) by overlap polymerase chain reaction (PCR) [19]. The resulting DNA fragment was inserted into the pHIL-S1 vector using the In-Fusion HD Cloning Kit (Clontech, CA, USA). The resultant plasmid was named pHIL-proROL-mut1-mROL. For the cell surface display of mROL^WT^ and mROL^imp^, the genes encoding proROL-mROL and proROL-mut1-mROL were amplified using the primers F BglII-proROL (5'- GCCAGATCTGTTCCTGTTTCTGGTAAATCTGG -3') and R XhoI-mROL (5'- ACCCTCGAGCAAACAGCTTCCTTCGTTGATAAAG -3'), and inserted into the pUGD1 plasmid. A gene encoding proROL-mut2-mROL was constructed using the protocol described for the proROL-mut1-mROL and then inserted into the pUGD1 plasmid. proROL-mut2-mROL encoded another mutated proROL, in which residues Gln50–Glu57 were replaced with eight Asn residues, and mROL. The resulting pUGD1-based plasmid was composed of the *GAPDH* promoter, secretion signal sequence of the glucoamylase gene, *ROL* gene, FLAG tag-encoding gene, and 3’-half of α-agglutinin gene.

### Production and purification of His-tagged mROL and mROL^imp^

pHIL-proROL-mROL, pHIL-proROL-mut1-mROL, and pHIL-S1 were digested with the restriction enzyme, *Sac*I. *P*. *pastoris* GS115 cells were transformed with the linear plasmids using the Frozen-EZ Yeast Transformation II kit (Zymo Research, CA, USA). The *P*. *pastoris* transformants were grown in 10 mL of BMGY medium for 24 h at 30°C. The culture was subsequently centrifuged at 3000 ×*g* for 5 min. The cells were resuspended in BMMY medium and then grown for 24 h at 30°C for protein production. All purification steps were performed at 4°C. The supernatant of the culture was collected at 3000 ×*g* for 5 min and concentrated using a YM-10 filter device (Millipore, MA, USA). ROL proteins were purified from the concentrated supernatant using the Ni Sepharose High Performance medium (GE Healthcare, Little Chalfont, UK), according to manufacturer’s protocol. To remove propeptide-uncleaved precursors, the purified fraction was mixed with an Anti-c-Myc-Agarose Affinity Gel (Sigma-Aldrich, MO, USA) for 3 h. Because c-Myc tags were fused to the N-terminus of the wild type and mutated propeptides, propeptide-uncleaved precursors were removed by this process and only mature forms (mROL^WT^ and mROL^imp^) were isolated. mROL^WT^ and mROL^imp^ were eluted by washing the resin four times with 500 μL of 50 mM potassium phosphate buffer (pH 6.5). Protein concentration was quantified using a Protein Assay Bicinchoninate Kit (Nacalai Tesque, Kyoto, Japan), which is based on Lowry assay [20].

### SDS-PAGE and N-terminal sequencing

The purity and molecular weights of the isolated proteins were confirmed by SDS-PAGE with or without EndoH (New England Biolabs, MA, USA) treatment on a 5–20% gradient polyacrylamide gel. Protein bands were detected using the CBB Stain One kit (Nacalai Tesque). Amino acid sequencing of the purified mROL^WT^ and mROL^imp^ was performed by the Edman degradation method on a protein sequencing system PPSQ-33A (Shimadzu, Kyoto, Japan), using a Hybond-P membrane (GE Healthcare).

### Activity assay

*p*-Nitrophenyl esters (2.5 mM) were used to determine the activities and substrate specificities of mROL^WT^ and mROL^imp^. *p*-Nitrophenyl esters (*p*-nitrophenyl acetate [C2], *p*-nitrophenyl butyrate [C4], *p*-nitrophenyl hexanoate [C6], *p*-nitrophenyl caprylate [C8], *p*-nitrophenyl decanoate [C10], *p*-nitrophenyl laurate [C12], *p*-nitrophenyl myristate [C14], and *p*-nitrophenyl palmitate [C16]) were dissolved in 50 mM potassium phosphate buffer (pH 6.5) containing 0.5% (v/v) Triton X-100 and emulsified using an ultrasonic homogenizer (output 100 W) for 1 min at room temperature. Purified mROL^WT^ and mROL^imp^ were diluted to a concentration of 10 μg/mL, and 2 μL of the ROL solution was added to 98 μL of substrate solutions. After incubation for 30 min at 30°C, lipase activity was estimated by measuring the absorbance at 405 nm. For the competitive lipase activity assays with peptidase substrates, 4 μL of 10 mM Suc-Ile-Ile-Trp-MCA (Peptide Institute, Osaka, Japan), dissolved in dimethyl sulfoxide, was added to 94 μL of 2.5 mM *p*-nitrophenyl butyrate. After the addition of 2 μL of lipase solution (10 μg/mL), the mixtures were incubated for 40 min at 30°C and the absorbance was measured at 405 nm. *p*-Nitrophenyl butyrate was used, because its high solubility ensured the better reproducibility. Kinetic parameters were calculated from a Hanes-Woolf plot, using 4-methylumbelliferyl butyrate, which is a sensitive substrate to lipase activities and soluble at broad concentrations (10–300 μM). The activation energy was determined using the Arrhenius equation, *E*_a_ = − *RT* ln(*k/A*), where A is the frequency factor, R is the universal gas constant (8.314 J·K^−1^·mol^−1^), T is the temperature, and *k* is the *k*_cat_ at each temperature.

### Determination of thermal stability and CD spectroscopy

The CD spectrum in the far-UV region (200–260 nm) was measured in a cell with an optical path length of 0.1 cm at 25°C using a JASCO J-720W spectropolarimeter (JASCO, Tokyo, Japan). The concentration of the protein was 50 μg/mL in 50 mM potassium phosphate buffer (pH 6.5). The fractions folded of mROL^WT^ and mROL^imp^ were measured by monitoring the negative ellipticities at 222 nm.

### Cell surface display of mROL^WT^, mROL^imp^, and mROL^imp2^

*S*. *cerevisiae* strain BY4741/*sed1*Δ was transformed with the above-mentioned plasmids using the Frozen-EZ Yeast Transformation II kit. Transformants displaying mROL^WT^, mROL^imp^, or mROL^imp2^ were grown in 5 mL of SD medium containing the appropriate amino acids for 24 h at 30°C. The cells were harvested and resuspended in the same medium. After cultivation for 24 h at 30°C, the cells were used for immunofluorescence labeling and activity assays. For the immunofluorescence labeling, the cells were incubated with an anti-FLAG M2 mouse monoclonal antibody (Sigma-Aldrich) at room temperature for 1 h. The cells were then washed with phosphate buffer saline (pH 7.4) and mixed with the secondary antibody (Alexa Fluor-488 goat anti-mouse IgG [Life Technologies]) for 1 h at room temperature. After washing, fluorescence was measured at the excitation (λ_ex_) and emission (λ_em_) wavelengths of 485 nm and 527 nm, respectively, using a Fluoroskan Ascent FL system (Labsystems, Helsinki, Finland). For the lipase activity assay, the cells were washed with 50 mM potassium phosphate buffer (pH 6.5) and the suspension was adjusted to OD_600_ of 3.0. After the addition of the substrate (100 μL) to the cell suspension (100 μL), the mixture was incubated for 20 min at 30°C with gentle shaking. For the peptidase activity assay, the cell suspension was adjusted to an OD_600_ of 20, and the cells (980 μL) were incubated with 10 mM Suc-Ile-Ile-Trp-MCA (20 μL) for 20 min at 30°C with gentle shaking. The amount of 7-amino-4-methylcoumarin was estimated at the excitation (λ_ex_) and emission (λ_em_) wavelengths of 355 nm and 460 nm, respectively.

## Results

### Production and purification of mROL^WT^ and mROL^imp^

**Fig 1:**
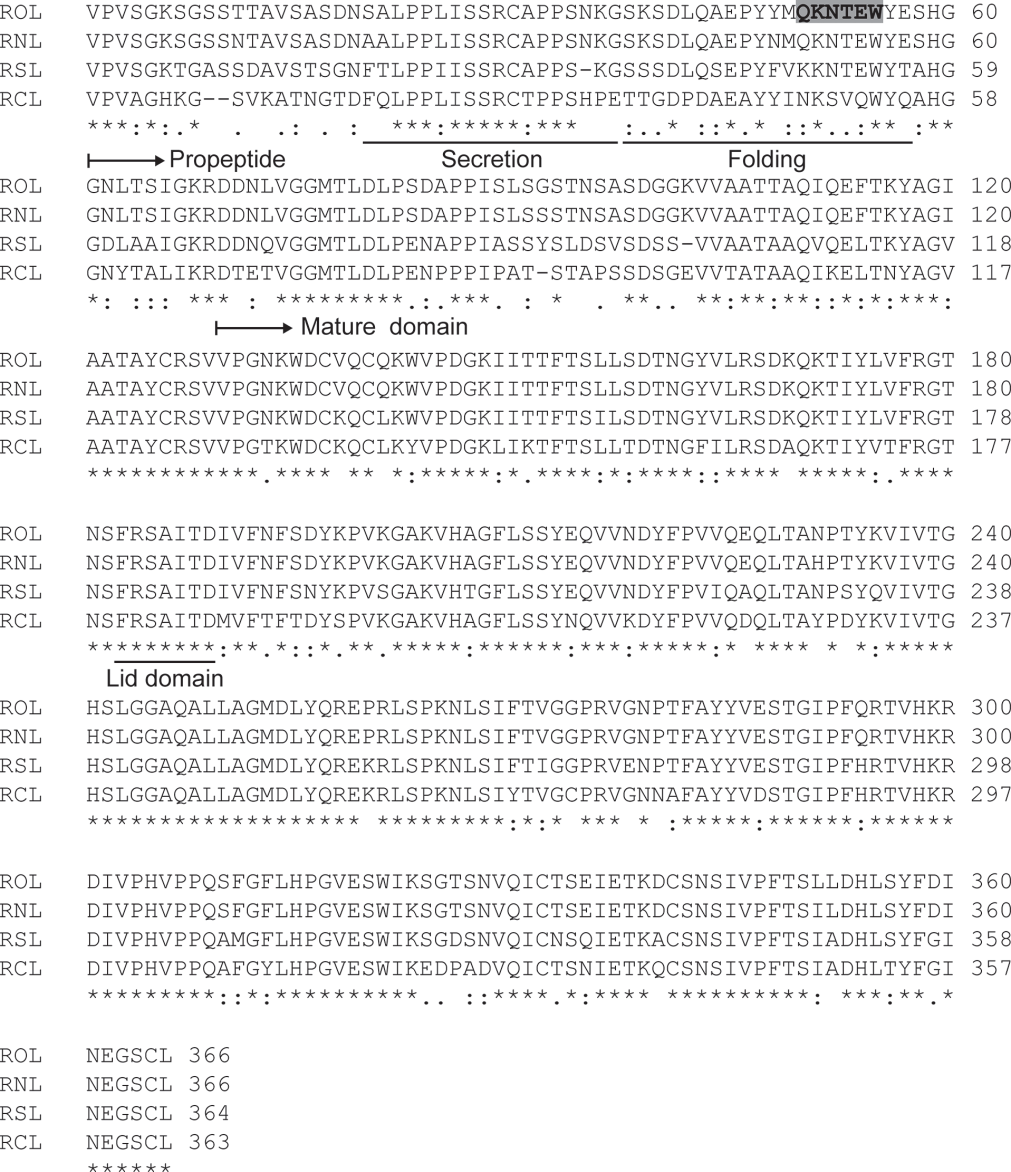
Sequence alignment of *Rhizopus oryzae* lipase (ROL) and ROL-related lipases. The full-length primary sequences of ROL, *R*. *niveus* lipase (RNL), *R*. *stolonifer* lipase (RSL), and *R*. *chinensis* lipase (RCL) are presented. Multiple-sequence alignments were generated using the ClustalW program (http://www.ebi.ac.uk/Tools/msa/clustalw2/). The underlined sequences in the propeptide of ROL (Ser20–Gly37 and Ser38–Glu57) indicate the regions that are essential for secretion and folding of mROL, respectively. The underlined sequences in the mature domain of ROL (Phe183–Asp189) indicate the lid domain. The shadowed region indicates residues that were replaced with hydrophilic amino acids (VDDDDK). In the original host, *R*. *oryzae*, the propeptide is also cleaved between the Ala97 and Ser98 residues [21]; however in *P*. *pastoris* and *S*. *cerevisiae*, the secondary cleavage has not been observed [14, 22]. Therefore, in this study, we defined the propeptide domain as the region between residues 1 and 69 and the mature domain as the region between residues 70 and 366 of ROL.

Our previous study revealed that residues Ser38–Glu57 of proROL were essential for the folding of mROL [6]. This region is highly conserved among 4 homologous lipases (Fig 1), and contains three charged amino acids (Asp41, Glu45, and Lys51) and many hydrophilic residues, indicating that proROL interacts with mROL via electrostatic and hydrophilic interactions. In particular, residues Gln50–Trp55 are highly hydrophilic. We replaced these residues with a more hydrophilic and charged amino acid sequence (VDDDDK) to alter the chaperoning function of this region. The mutated propeptide was named proROL-mut1 and the mature ROL folded by proROL-mut1 was named mROL^imp^ (imprinted ROL) to distinguish it from mROL^WT^ (mROL folded by wild type proROL). The two distinct mROL^WT^ and mROL^imp^ with the same primary sequence were produced and purified from *P*. *pastoris* (S1 Fig) using a two-step purification protocol (see Materials and Method). No apparent decrease in molecular weight was observed after endoglycosidase H (EndoH) treatment, as analyzed by sodium dodecyl sulfate-polyacrylamide gel electrophoresis (SDS-PAGE), indicating that mROL^WT^ and mROL^imp^ were not *N*-glycosylated. To confirm that proROL and proROL-mut1 were cleaved at the same site and that the primary sequences of mROL^WT^ and mROL^imp^ were identical, the protein bands were excised and analyzed by N-terminal sequencing. The analysis confirmed that both sequences of mROL^WT^ and mROL^imp^ started with DDNLVGGMTLD, indicating that the propeptides were cleaved at the same site and that the primary sequences of mROL^WT^ and mROL^imp^ were the same.

### Lipase activity assay

**Fig 2:**
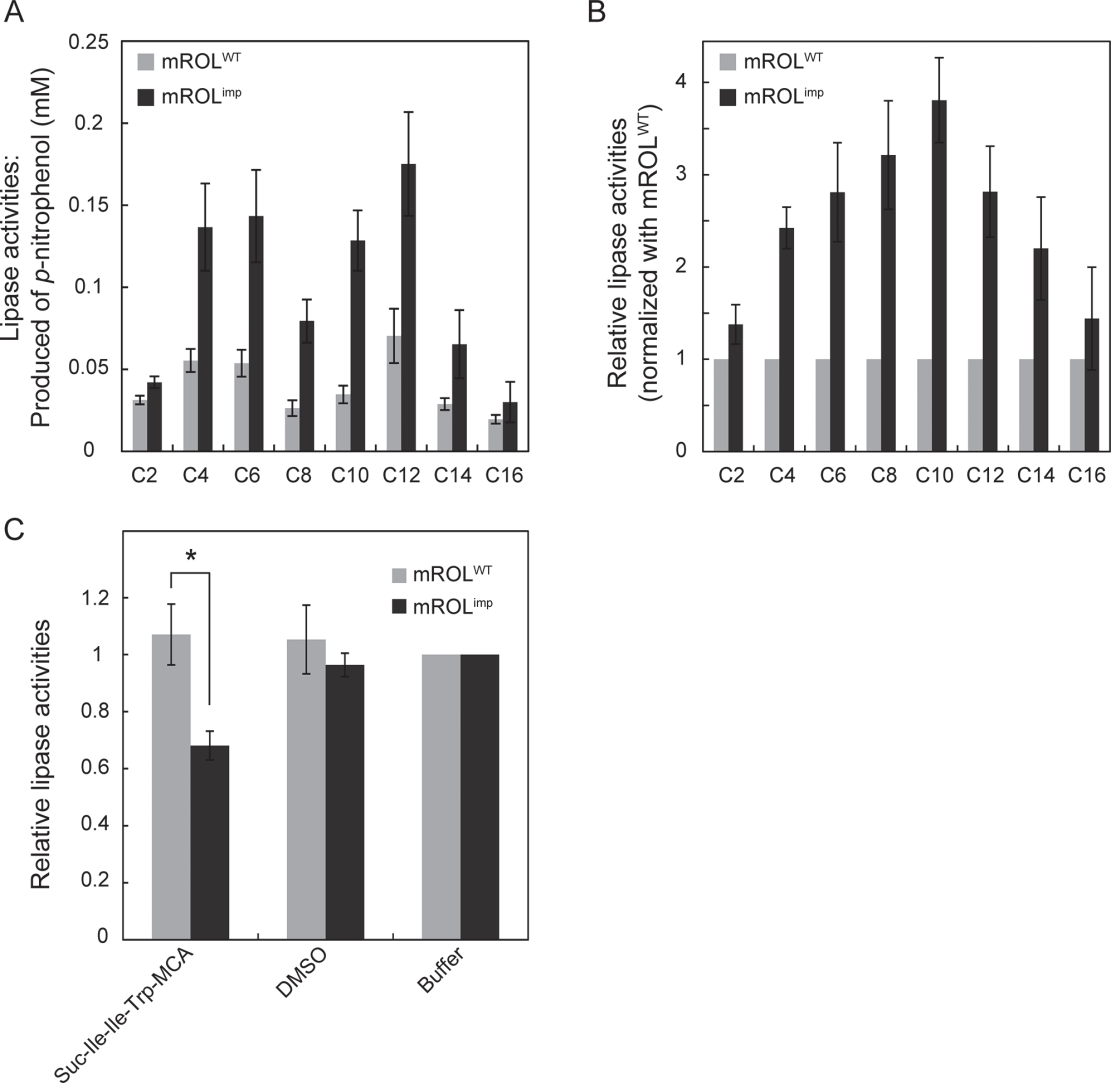
Lipase activity assay with purified ROL. (A) Measurement of the lipase activities of mROL^WT^ and mROL^imp^ using *p*-nitrophenyl esters with various acyl chain lengths (C2–C16) as substrates. The resultant *p*-nitrophenol was quantified to estimate the lipase activities. (B) Relative lipase activities normalized with the values obtained for mROL^WT^. The values are presented as mean ± standard error of the mean (SEM) based on at least three independent measurements. (C) Competitive lipase activity assay with purified mROL^WT^ and mROL^imp^. Lipase activities were determined in the presence of the peptidase substrate, Suc-Ile-Ile-Trp-MCA, dissolved in dimethyl sulfoxide (DMSO). The *P*-values were determined using the Student’s *t*-test. * *P* < 0.05.

The activities of mROL^WT^ and mROL^imp^ were measured using *p*-nitrophenyl esters as substrates to investigate their substrate specificities toward acyl groups of different chain lengths (C2–C16). Replacement of the hydrophilic residues of proROL with alternative residues had a considerable effect on the chaperoning function of the propeptide. The activity of mROL^imp^ was higher than that of mROL^WT^, despite the fact that mROL^imp^ and mROL^WT^ had the same primary sequence (Fig 2A). The mutated propeptide not only generated mature protein with a higher activity but also altered the chain-length specificity. Compared with mROL^WT^, mROL^imp^ showed a preference for substrates with medium-chain length acyl groups (Fig 2B). In particular, mROL^imp^ exhibited 3.8-fold higher activity toward *p*-nitrophenyl decanoate (C10), and only 1.4-fold higher activity toward *p*-nitrophenyl palmitate (C16). To further characterize mROL^imp^, the enzyme kinetics of mROL^WT^ and mROL^imp^ were evaluated using 4-methylumbelliferyl butyrate (Table 1). The *K*_M_ value of mROL^imp^ was higher than that of mROL^WT^, indicating that the substrate specificity had been altered by proROL-mut1, resulting in a lower affinity of mROL^imp^ for the substrate. However, mROL^imp^ exhibited a significantly higher *k*_cat_ value than mROL^WT^. The activation energy of mROL^imp^ was also lower than that of mROL^WT^. These results suggest that the mutated propeptide affected the conformation of mROL^imp^ not only around the substrate-binding site but also around the active site. A comparison of the activation energies indicated that the higher turnover rate of mROL^imp^ than mROL^WT^ may be attributed to the lower activation energy.

**Table 1:** Kinetic parameters for mROL and mROL^imp^.

	*K*_M_ (μM)	*k*_cat_ (s^-1^)	*k*_cat_/*K*_M_ (mM^-1^ s^-1^)	Ea (kJ mol^-1^)
mROL	24 ± 11	0.054 ± 0.003	2.9 ± 0.9	79 ± 10
mROL^imp^	79 ± 5	0.15	1.9 ± 0.2	46 ± 6
*P*-value	6.1 × 10^–3^	2.6×10^–5^	0.82	4.9×10^–2^

Results are show as mean ±sem.

### Altered Substrate recognition

In order to further characterize mROL^imp^, we investigated whether the altered substrate binding site allowed mROL^imp^ to recognize an alternative substrate that mROL^WT^ could not recognize. The active sites of most lipases, including ROL, are mainly composed of three residues, Asp, His, and Ser [15, 23, 24]. These residues are also found in the active sites of serine proteases and peptidases [25]. Most serine protease and peptidases can be classified into three groups: type I, type II, and type III [26]. The disposition of the residues in the active site of ROL resembles that of type III serine protease (carboxypeptidase Y) closely (S2 Fig). Therefore, we investigated whether mROL^WT^ and mROL^imp^ recognized a peptidase-specific substrate by performing a lipase activity assay in the presence of a peptidase-specific substrate as a competitor. We used Suc-Ile-Ile-Trp-MCA as the peptidase-specific substrate; Suc-Ile-Ile-Trp-MCA is a hydrophobic substrate that is effectively cleaved by the type III serine peptidase, CPY [9]. Intriguingly, the peptidase substrate competed with the lipase substrate (*p*-nitrophenyl butyrate) for mROL^imp^, although it did not compete with the lipase substrate in the case of mROL^WT^ (Fig 2C). This result further suggests that the mutated propeptide altered the substrate-binding site and allowed mROL^imp^ to recognize the peptidase substrate.

### Structural imprinting

**Fig 3:**
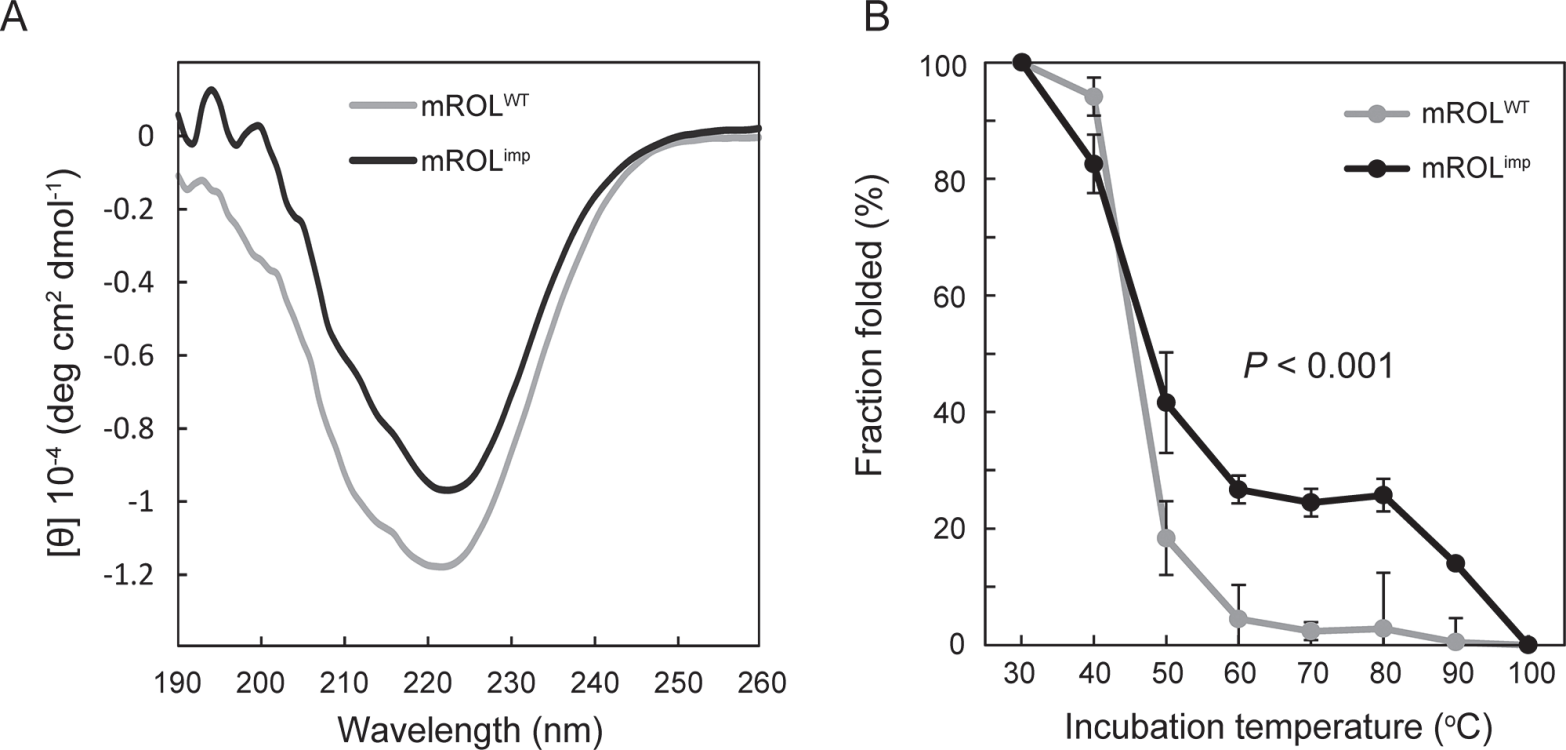
Analysis of the structures and stabilities of mROL^WT^ and mROL^imp^. (A) Circular dichroism spectra of mROL^WT^ and mROL^imp^. (B) The thermal stabilities of mROL^WT^ and mROL^imp^. The negative ellipticities at 222 nm were measured to determine the fraction folded. The values are presented as mean ± SEM based on three independent measurements. The *P*-value was determined using a two-factor ANOVA with mROLs and temperatures as independent factors.

We hypothesized that the mutated propeptide differently folded mROL and generated mROL^imp^ which had distinct activities and substrate recognitions. The folding information of mROL^imp^ were investigated using a far-ultraviolet (UV) circular dichroism (CD) spectrum. CD spectrum revealed that mROL^imp^ obtained different secondary structures from mROL^WT^ (Fig 3A). In addition, mROL^imp^ was significantly more stable than mROL^WT^ at higher temperatures (Fig 3B), according to the changes in negative ellipticity at 222 nm at each temperature. In contrast to mROL^WT^, mROL^imp^ exhibited an intermediate state in the process of denaturation (60–80°C), indicating that the different denaturation pattern was attributed to the distinct secondary structure of mROL^imp^. The altered folding function of proROL-mut1 resulted in the generation of mROL^imp^ which improved the lipase activities, altered the substrate recognitions, and had higher stability, in spite of having the same amino acid sequences as mROL^WT^.

## Discussion

In this study, we generated a functionally distinct mROL by mutating the propeptide of ROL. The mutated propeptide produced a distinct mROL^imp^ with higher activity, and altered substrate recognition compared with mROL^WT^, even though the amino acid sequences of both lipases were the same. In addition, the structural stability of mROL^imp^ was also improved compared with mROL^WT^. Because of the altered chaperoning function of proROL-mut1, mROL^imp^ would be folded via an alternative folding pathway and reach a distinct minimum in the folding free energy landscape. The phenomenon of “protein folding memory” implies that proteins are not always folded into their most stable states, but can be folded into various states depending on their folding circumstances. “Protein folding memory” has also observed in CPY [9] and subtilisin (type II serine protease) [27, 28]. In these studies, mature domains were also folded into distinct states depending on their mutated propeptide. In this point, “protein folding memory” contradicts Anfinsen’s dogma.

**Fig 4:**
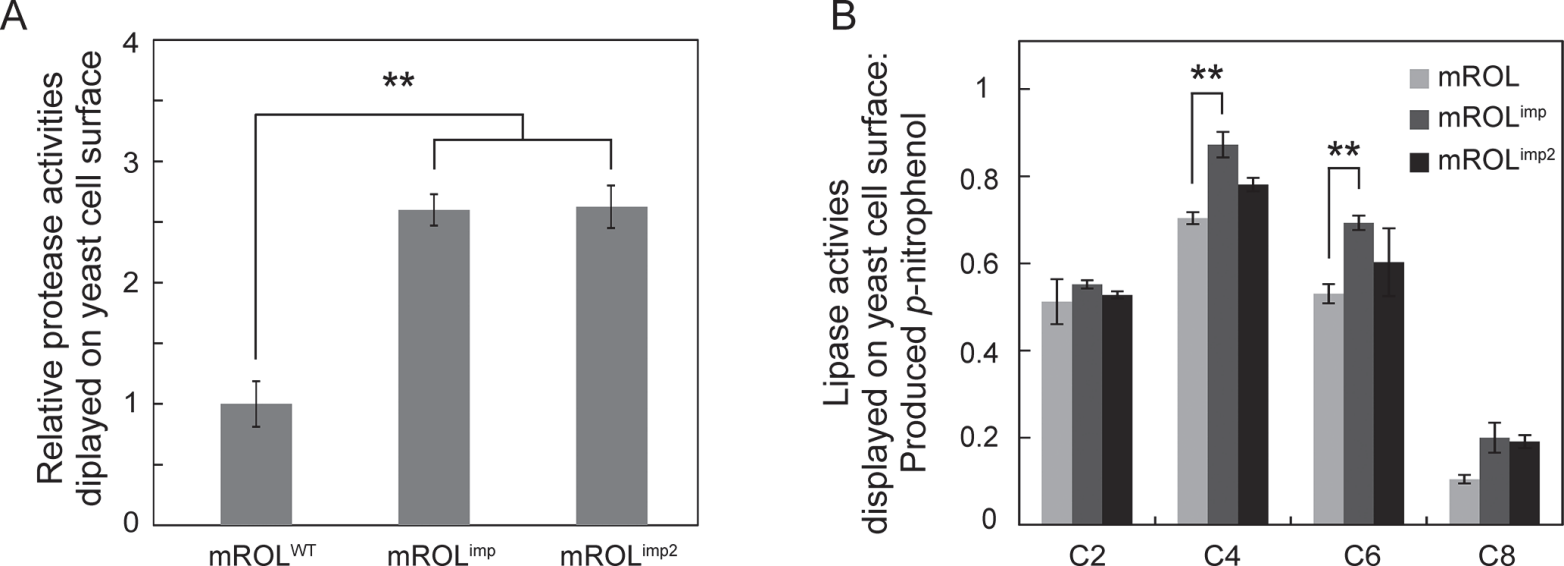
Activity assay using yeast cell surface engineering. (A) Peptidase activity assay of mROL^WT^, mROL^imp^, and mROL^imp2^ displayed on the yeast cell surface. (B) Lipase activity assay of mROL^WT^, mROL^imp^, and mROL^imp2^ displayed on the yeast cell surface. The resultant *p*-nitrophenol was quantified to estimate the lipase activities. The peptidase and lipase activities were corrected by the number of displayed enzymes (S3 Fig). The values are presented as mean ± SEM based on three independent measurements. The *P*-values were determined using one-way analysis of variance followed by Tukey’s test for multiple comparisons. ** *P* < 0.01.

The results of the lipase activity assay in the presence of peptidase-specific substrates revealed that purified mROL^imp^ recognized the peptidase-specific substrate, while mROL^WT^ did not. However, neither mROL^WT^ nor mROL^imp^ exhibited any peptidase activity. As an alternative method to evaluate peptidase activity, we adopted a cell-surface engineering technique, using the yeast *Saccharomyces cerevisiae* [29]. This method allows the display of a number of proteins on the cell surface of yeast and enables a simple and rapid activity assay. The productions and displays of mROL^WT^ and mROL^imp^ were confirmed by immunofluorescence labeling (S3 Fig). Remarkably, mROL^imp^ displayed on the yeast cell surface exhibited significantly higher peptidase activity than mROL^WT^ (Fig 4A). The displayed mROL^imp^ also showed higher lipase activities, similar to that of purified mROL^imp^ (Fig 4B). The mutated propeptide potentiated peptidase activity for mROL and immobilization on the cell wall enabled mROL^imp^ to cleave the peptidase substrate.

**Fig 5:**
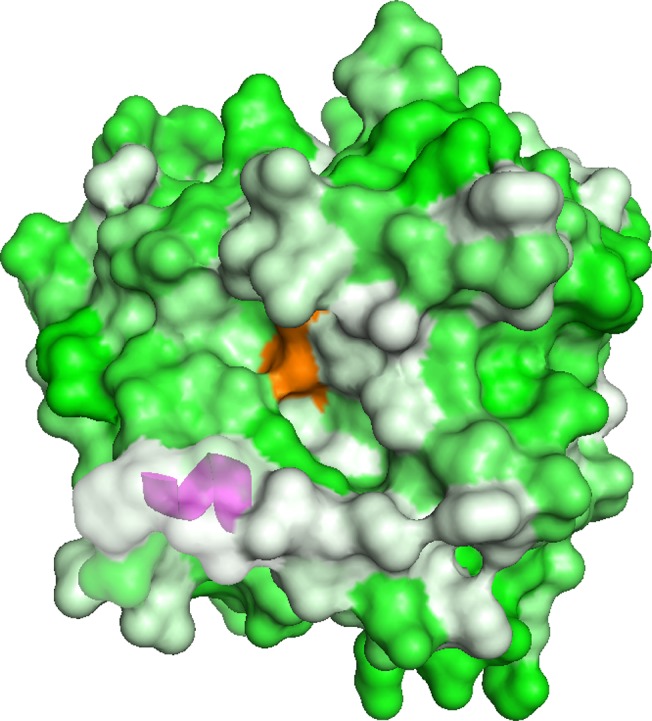
Structure of mROL^WT^, modeled using the SWISS-MODEL program. The structure of mROL^WT^ was modeled based on the open-lid structure of *Rhizomucor miehei* lipase (Protein Data Bank [PDB] ID: 4TGL), and visualized using PyMOL. The active site residues, S242, D301, and H354, are colored orange. The magenta-colored α-helix represents the lid domain. Green residues indicate hydrophilic amino acids and white residues indicate hydrophobic amino acids.

Most lipases have a lid domain that conceals the active site [16, 17]. In the case of *Rhizomucor miehei* lipase, which has 50% sequence homology to ROL, an open lid that forms a hydrophobic groove is important for substrate recognition [16]. The tertiary structure of mROL^WT^, modeled from the open-lid structure of *R*. *miehei* lipase, revealed that the open lid of mROL^WT^ did not form a hydrophobic groove around the active site (Fig 5). The mutated propeptide probably affected the conformation of the lid domain and allowed the open lid to form a hydrophobic groove to interact with substrates composed of medium-chain length acyl groups and a hydrophobic peptidase-specific substrate. A previous study showed that mutations in the lid domain changed the substrate specificity of mROL [17], indicating that the open lid plays a critical role in substrate recognition. Because the lid domain and surrounding residues in ROL contain hydrophilic residues such as Arg184, Ser185, Thr188, and Asp189 (Fig 1), the additional hydrophilic residues introduced in the mutated propeptide might interact with the residues of the lid domain via hydrogen bonding. Interestingly, another propeptide mutant in which residues Gln50–Glu57 (which are essential for folding) were replaced with eight hydrophilic Asn residues also exhibited “protein folding memory” and induced peptidase activity in the mature domain of ROL (named mROL^imp2^; Fig 4A and 4B). These results strongly suggest that the hydrophilic residues in the propeptide interacted with the lid domain and surrounding residues in a different manner compared with wild type propeptide. CD spectrum analyses showed change in the secondary structure and thermal stability of mROL^imp^; however, it did not reveal detailed folding mechanisms of mROL^WT^ and mROL^imp^. Crystallization analyses of mROL^WT^ and mROL^imp^ will provide further insight into the phenomenon of “protein folding memory”. Although we have attempted to crystallize ROL according to the crystallization methods for homologous lipases such as *R*. *niveus* lipase and *R*. *miehei* lipase, but it has been difficult. The elaborated structural characterizations of mROLs would be our subject of the future work.

Some peptide fragments have been reported to take both alpha- and beta- conformations depending on their context [30, 31]. Dalal et al. previously converted a predominantly β-sheet protein into four-helix bundle protein by redesigning the 50% of sequence in the original β-sheet protein [32]. Similarly, Alexander et al. designed two proteins that had 88% homology and completely distinct structures [33] and suggested that only 12% of the amino acids in the protein determined the whole structure. These studies clearly demonstrate that folding information for a protein structure does not reside equally in all residues in a protein, but rather resides in only a few residues. These studies, however, were limited to the folding information in mature domains. Our study further extends the idea. We suggest that the folding information is contained not only in a mature domain, but also in a propeptide domain which is cleaved off during maturation steps. In “protein folding memory”, the mature domain folds depending on the folding information in the propeptide and retains its folding memory even after the cleavage of the propeptide.

In conclusion, we demonstrated that mutated proROL exhibited “protein folding memory” and generated a functionally distinct and structurally more stable mROL^imp^ that had the same amino acid sequence as mROL^WT^.

## Supporting Information

S1 Fig SDS-PAGE analysis with and without endoglycosidase H (EndoH) treatment. *N*-glycosylation was not observed. *, mature form of ROL; **, EndoH; M, marker.
(TIF)

S2 Fig Active sites of ROL and proteases. The active site of ROL is composed of three residues, S242, D301, and H354. These residues also form the catalytic triad in the active sites of serine proteases, bovine pancreas trypsin (PDB: 1S0Q), subtilisin (PDB: 2SIC), and carboxypeptidase Y (CPY; PDB: 1YSC). The steric conformation of the active site in ROL is particularly similar to that of the type III serine protease, CPY.
(TIF)

S3 Fig Immunofluorescence labeling of the displayed mROLs. (A) The displays of mROL^WT^, mROL^imp^, and mROL^imp2^ were confirmed by immunofluorescence labeling. Positive control indicates yeast cells displaying only the FLAG tag and negative control indicates yeast cells displaying the strep-tag instead of the FLAG tag. Scale bar, 5 μm. (B) The number of displayed proteins was quantified by measuring fluorescence at the excitation (λ_ex_) and emission (λ_em_) wavelengths of 355 and 460 nm, respectively.
(TIF)

## References

[1] Ikemura H, Takagi H, Inouye M. Requirement of pro-sequence for the production of active subtilisin E in *Escherichia coli* . J Biol Chem. 1987; 262: 7859–7864. 3108260

[2] Inouye M. Intramolecular chaperone: the role of the pro-peptide in protein folding. Enzyme. 1990; 45: 314–321. 10.1159/000468904 1688202

[3] Takahashi S, Ueda M, Atomi H, Beer HD, Bornscheuer UT, Schmid RD, et al Extracellular production of active *Rhizopus oryzae* lipase by *Saccharomyces cerevisiae* . J Ferment Bioeng. 1998; 86: 164–168.

[4] Boel E, Huge-Jensen B, Christensen M, Thim L, Fiil NP. *Rhizomucor miehei* triglyceride lipase is synthesized as a precursor. Lipids. 1988; 23: 701–706. 3419283 10.1007/BF02535672

[5] Takahashi S, Ueda M, Tanaka A. Independent production of two molecular forms of a recombinant *Rhizopus oryzae* lipase by *KEX2*-engineered strains of *Saccharomyces cerevisiae* . Appl Microbiol Biotechnol. 1999; 52: 534–540. 10570801 10.1007/s002530051556

[6] Takahashi S, Ueda M, Tanaka A. Function of the prosequence for *in vivo* folding and secretion of active *Rhizopus oryzae* lipase in *Saccharomyces cerevisiae* . Appl Microbiol Biotechnol. 2001; 55: 454–462. 11398926 10.1007/s002530000537

[7] Ramos C, Winther JR, Kielland-Brandt M. Requirement of the propeptide for *in vivo* formation of active yeast carboxypeptidase Y. J Biol Chem. 1994; 269: 7006–7012. 8120064

[8] Susanne OS, Van Den Hazel MC, Kielland-Brandt MC, Winther JR. pH-dependent processing of yeast procarboxypeptidase Y by proteinase A *in vivo* and *in vitro* . Eur J Biochem. 1994; 220: 19–27. 8119286 10.1111/j.1432-1033.1994.tb18594.x

[9] Nagayama M, Maeda H, Kuroda K, Ueda M. Mutated intramolecular chaperones generate high-activity isomers of mature enzymes. Biochemistry. 2012; 51: 3547–3553. doi: 10.1021/bi3001159 22482366 10.1021/bi3001159

[10] Anfinsen CB. The formation and stabilization of protein structure. Biochem J. 1972; 128: 737–749. 4565129 10.1042/bj1280737 PMC1173893

[11] Ghosh B, Ray RR. Current commercial perspective of *Rhizopus oryzae*: a review. J Appl Sci. 2011; 11: 2470–2486.

[12] Haas MJ, Bailey DG, Baker W, Berka TR, Cichowicz DJ, Derewenda ZS, et al Biochemical and molecular biological characterization of a lipase produced by the fungus *Rhizopus delemar* . Eur J Lipid Sci Tech. 1999; 101: 364–369.

[13] Beer HD, Wohlfahrt G, Schmid RD, McCarthy JE. The folding and activity of the extracellular lipase of *Rhizopus oryzae* are modulated by a prosequence. Biochem J. 1996; 319: 351–359. 8912667 10.1042/bj3190351 PMC1217776

[14] Minning S, Schmidt-Dannert C, Schmid RD. Functional expression of *Rhizopus oryzae* lipase in *Pichia pastoris*: high-level production and some properties. J Biotechnol. 1998; 66: 147–156. 9866866 10.1016/s0168-1656(98)00142-4

[15] Shibamoto H, Matsumoto T, Fukuda H, Kondo A. Molecular engineering of *Rhizopus oryzae* lipase using a combinatorial protein library constructed on the yeast cell surface. J Mol Catal B: Enzym. 2004; 28: 235–239.

[16] Brzozowski AM, Derewenda U, Derewenda ZS, Dodson GG, Lawson DM, Turkenburg JP, et al A model for interfacial activation in lipases from the structure of a fungal lipase-inhibitor complex. Nature. 1991; 351: 491–494. 2046751 10.1038/351491a0

[17] Shiraga S, Ishiguro M, Fukami H, Nakao M, Ueda M. Creation of *Rhizopus oryzae* lipase having a unique oxyanion hole by combinatorial mutagenesis in the lid domain. Appl Microbiol Biotechnol. 2005; 68: 779–785. 15729555 10.1007/s00253-005-1935-0

[18] Derewenda U, Brzozowski AM, Lawson DM, Derewenda ZS. Catalysis at the interface-the anatomy of a conformational change in a triglyceride lipase. Biochemistry. 1992; 31: 1532–1541. 1737010 10.1021/bi00120a034

[19] Horton RM, Cai Z, Ho S, Pease L. Gene splicing by overlap extension: tailor-made genes using the polymerase chain reaction. BioTechniques. 1990; 8: 528–535. 2357375

[20] Smith P, Krohn RI, Hermanson G, Mallia A, Gartner F, Provenzano M, et al Measurement of protein using bicinchoninic acid. Anal Biochem. 1985; 150: 76–85. 3843705 10.1016/0003-2697(85)90442-7

[21] Haas MJ, Allen J, Berka TR. Cloning, expression and characterization of a cDNA encoding a lipase from *Rhizopus delemar* . Gene. 1991; 109: 107–113. 1756969 10.1016/0378-1119(91)90594-2

[22] Ueda M, Takahashi S, Washida M, Shiraga S, Tanaka A. Expression of *Rhizopus oryzae* lipase gene in *Saccharomyces cerevisiae* . J Mol Catal B: Enzym. 2002; 17: 113–124.

[23] Derewenda ZS, Derewenda U, Dodson GG. The crystal and molecular-structure of the *Rhizomucor miehei* triacylglyceride lipase at 1.9-angstrom resolution. J Mol Biol. 1992; 227: 818–839. 1404390 10.1016/0022-2836(92)90225-9

[24] Emmerich J, Beg O, Peterson J, Previato L, Brunzell J, Brewer H, et al Human lipoprotein lipase. Analysis of the catalytic triad by site-directed mutagenesis of Ser-132, Asp-156, and His-241. J Biol Chem. 1992; 267: 4161–4165. 1371284

[25] Brady L, Brzozowski AM, Derewenda ZS, Dodson E, Dodson G, Tolley S, et al. A serine protease triad forms the catalytic centre of a triacylglycerol lipase. 1990. 10.1038/343767a0 2304552

[26] Robertus JD, Kraut J, Alden RA, Birktoft JJ. Subtilisin. Stereochemical mechanism involving transition-state stabilization. Biochemistry. 1972; 11: 4293–4303. 5079900 10.1021/bi00773a016

[27] Shinde UP, Liu JJ, Inouye M. Protein memory through altered folding mediated by intramolecular chaperones. Nature. 1997; 389: 520–522. 9333245 10.1038/39097

[28] Shinde U, Fu X, Inouye M. A pathway for conformational diversity in proteins mediated by intramolecular chaperones. J Biol Chem. 1999; 274: 15615–15621. 10336458 10.1074/jbc.274.22.15615

[29] Kuroda K, Ueda M. Arming technology in yeast—novel strategy for whole-cell biocatalyst and protein engineering. Biomolecules. 2013; 3: 632–650. doi: 10.3390/biom3030632 24970185 10.3390/biom3030632 PMC4030959

[30] Mezei M. Chameleon sequences in the PDB. Protein engineering. 1998; 11: 411–414. 9725618 10.1093/protein/11.6.411

[31] Ghozlane A, Joseph AP, Bornot A, de Brevern AG. Analysis of protein chameleon sequence characteristics. Bioinformation. 2009; 3: 367 19759809 10.6026/97320630003367 PMC2732029

[32] Dalal S, Balasubramanian S, Regan L. Protein alchemy: Changing beta-sheet into alpha-helix. Nat Struct Biol. 1997; 4: 548–552. 9228947 10.1038/nsb0797-548

[33] Alexander PA, He Y, Chen Y, Orban J, Bryan PN. The design and characterization of two proteins with 88% sequence identity but different structure and function. Proc Natl Acad Sci USA. 2007; 104: 11963–11968. 17609385 10.1073/pnas.0700922104 PMC1906725

