# Supplementary figures and images for: Generation of a Functionally Distinct Rhizopus oryzae Lipase through Protein Folding Memory
Source: PLoS One. 2015 May 13;10(5):e0124545. doi: 10.1371/journal.pone.0124545 (PMC4430139; doi:10.1371/journal.pone.0124545)

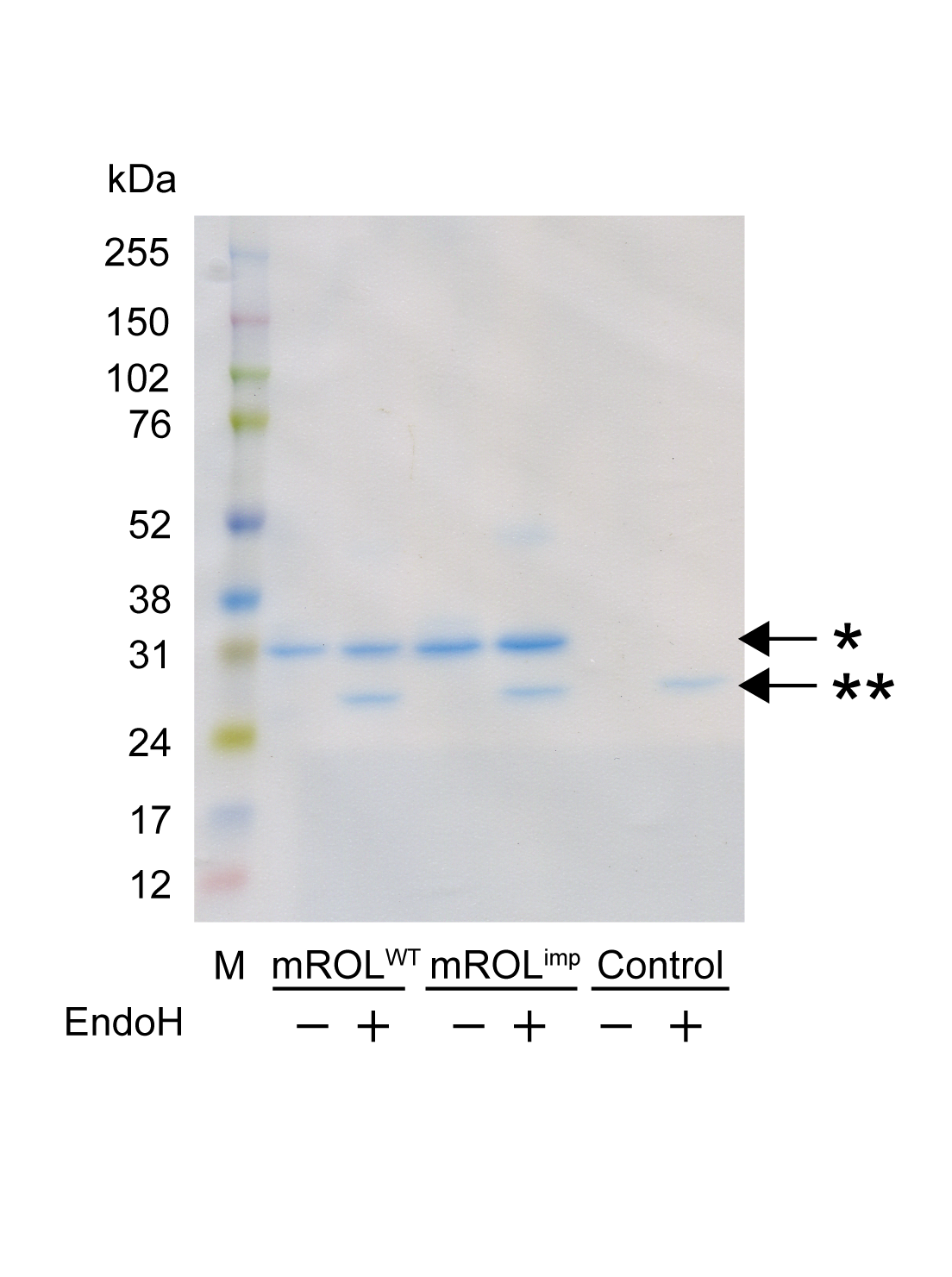

Supplement: S1 Fig — N-glycosylation was not observed. *, mature form of ROL; **, EndoH; M, marker. (TIF) [file pone.0124545.s001.tif]

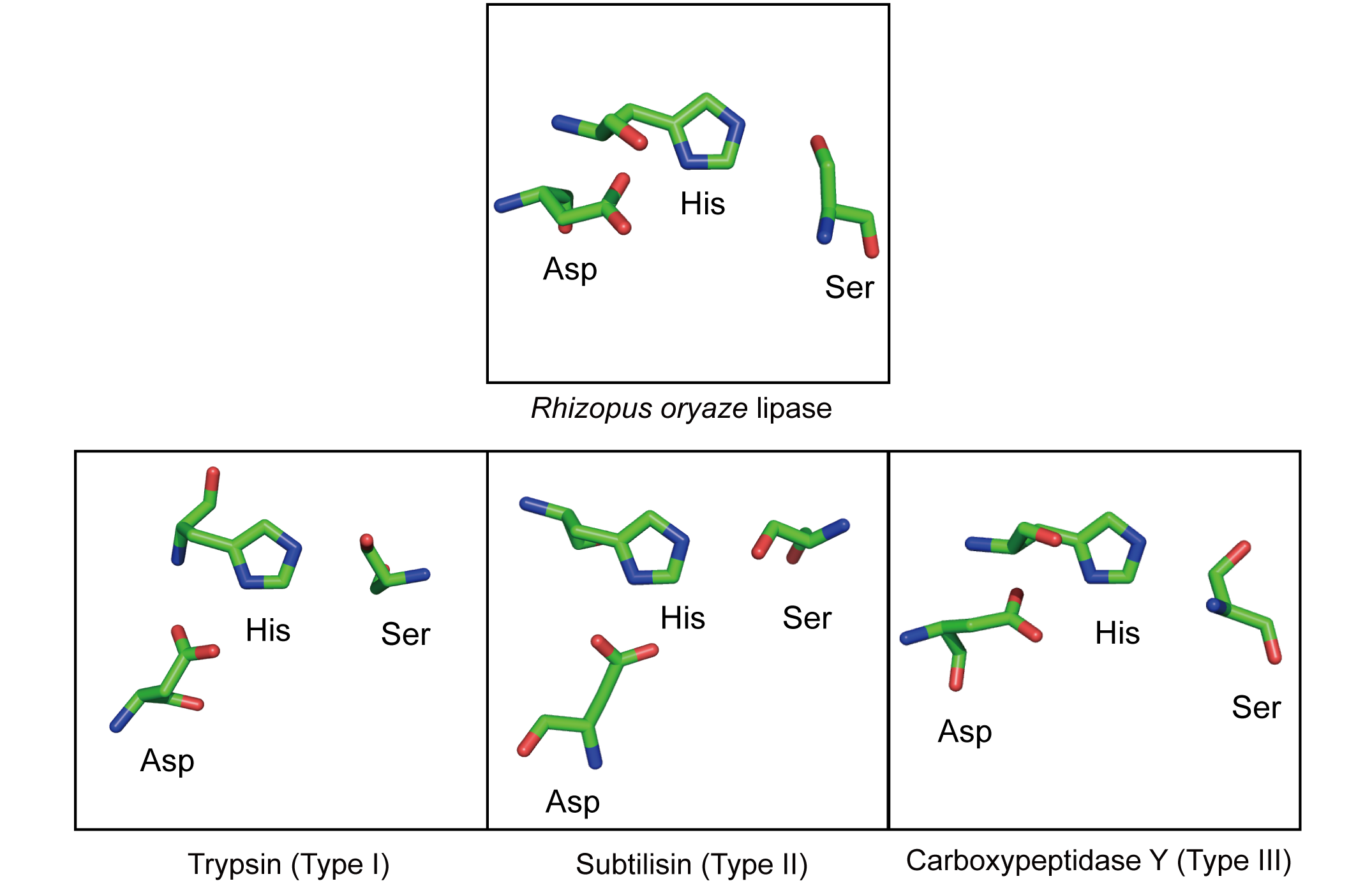

Supplement: S2 Fig — The active site of ROL is composed of three residues, S242, D301, and H354. These residues also form the catalytic triad in the active sites of serine proteases, bovine pancreas trypsin (PDB: 1S0Q), subtilisin (PDB: 2SIC), and carboxypeptidase Y (CPY; PDB: 1YSC). The steric conformation of the active site in ROL is particularly similar to that of the type III serine protease, CPY. (TIF) [file pone.0124545.s002.tif]

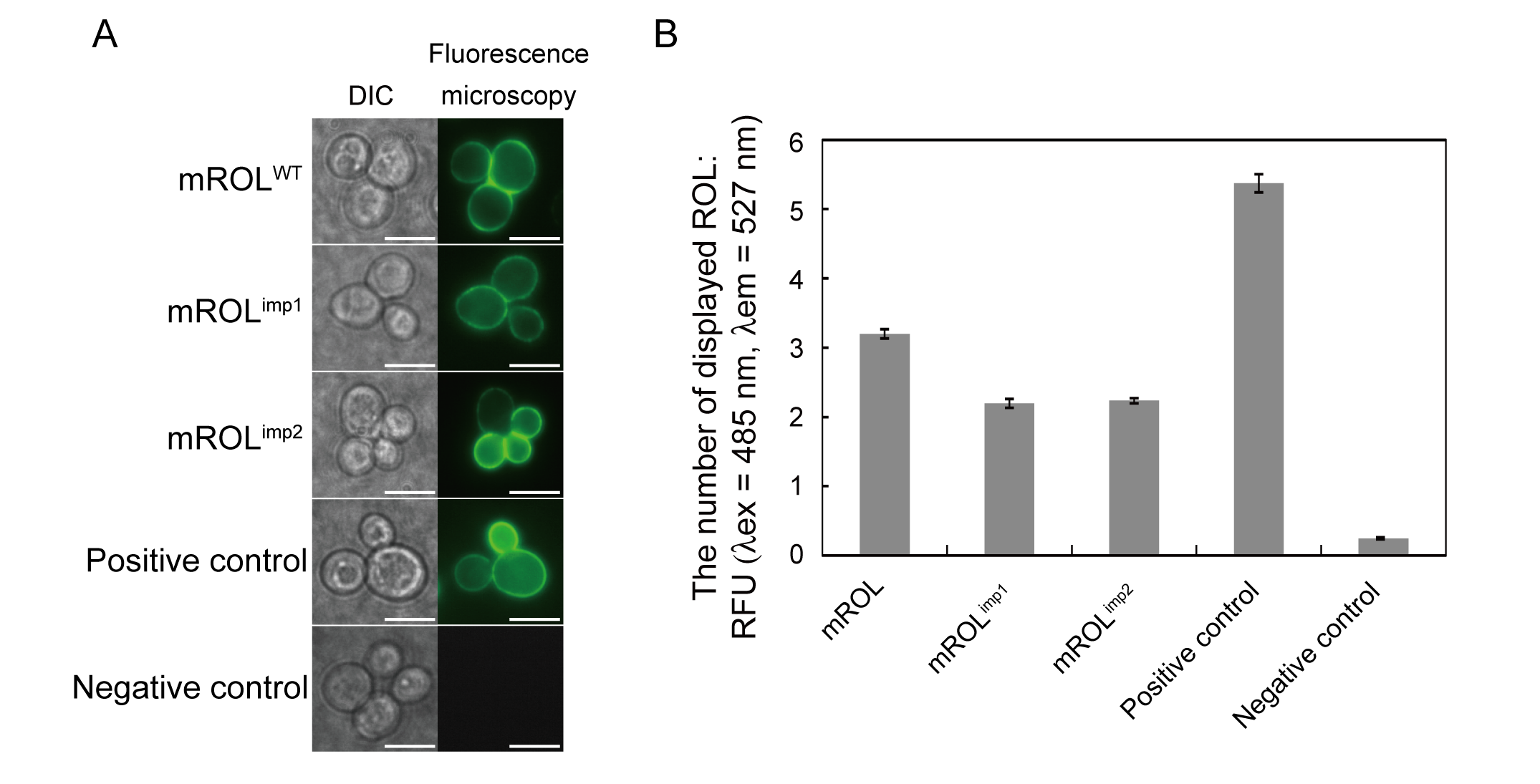

Supplement: S3 Fig — (A) The displays of mROLWT, mROLimp, and mROLimp2 were confirmed by immunofluorescence labeling. Positive control indicates yeast cells displaying only the FLAG tag and negative control indicates yeast cells displaying the strep-tag instead of the FLAG tag. Scale bar, 5 μm. (B) The number of displayed proteins was quantified by measuring fluorescence at the excitation (λex) and emission (λem) wavelengths of 355 and 460 nm, respectively. (TIF) [file pone.0124545.s003.tif]
